# Clinicopathological feature and prognosis of primary hepatic gastrointestinal stromal tumor

**DOI:** 10.1002/cam4.808

**Published:** 2016-08-03

**Authors:** Zhen Liu, Yangzi Tian, Shushang Liu, Guanghui Xu, Man Guo, Xiao Lian, Daiming Fan, Hongwei Zhang, Fan Feng

**Affiliations:** ^1^ Department of Digestive SurgeryXijing Hospital, The Fourth Military Medical University127 West Changle RoadXi'anShaanxi710032China; ^2^ Department of DermatologyXijing Hospital, The Fourth Military Medical University127 West Changle RoadXi'anShaanxi710032China

**Keywords:** Feature, gastrointestinal stromal tumor, liver, prognosis

## Abstract

Compared to gastric gastrointestinal stromal tumor (GIST), hepatic GIST is very rare in clinic. Reports on clinicopathological feature and prognosis of this rare disease are limited in literature. The purpose of this study was, therefore, to summarize clinical and pathological features as well as prognosis of the primary hepatic GIST. One case of primary hepatic GIST from our center and 22 cases reported in MEDLINE or China National Knowledge Infrastructure (CNKI) were enrolled into this study. Clinicopathological features as well as survival data of hepatic GIST were analyzed and compared with 297 gastric GISTs and 59 small intestinal GISTs from our center. Majority of the 22 cases (95.7%) of hepatic GIST was larger than 5 cm in size, and 75.0% of the tumors were over 5/50 HPF in mitotic index. Most of the hepatic GISTs (85.7%) displayed spindle cell shape in morphology. All of the hepatic GIST (100%) enrolled in this study were classified as high‐risk category by the National Institute of Health (NIH) risk classification. The 5‐year median disease‐free survival (DFS) time was 24.0 months and 5‐year disease‐specific survival (DSS) rate was 33.3%, respectively. Distribution of clinicopathological features was significantly different among hepatic, gastric, and small intestinal GIST. The DFS and DSS of hepatic GIST were significantly lower than those of the other two groups. Majority of the hepatic GIST is large in size and highly malignant. Prognosis of the primary hepatic GIST is worse than that of gastric GIST and small intestinal GIST.

## Introduction

Gastrointestinal stromal tumor (GIST) is the most common mesenchymal tumor in human gastrointestinal (GI) tract, which is considered to arise from the interstitial cells of Cajal (ICC) 10. Gain‐of‐function mutation of the receptor tyrosine kinase (RTK) gene, *KIT*, a stem cell factor receptor, plays a crucial role in the oncogenesis of most GISTs 6. In this content, 65–80% of GISTs have *KIT* gene mutation, and the other, approximately 10% of *KIT*‐negative GISTs have activating mutation in the gene of platelet‐derived growth factor receptor α (*PDGFRA*)^9^. The mutation of *KIT/PDGFRA* may contribute to the occurrence and development of GIST by leading to the activation and autophosphorylation of the downstream signaling pathways 28.

GIST accounts for nearly 2.2% of GI malignancies 35. Notably, approximately 60–70% of GIST occurs in the stomach, followed by 20–30% in small intestine, 5% in the colon and rectum, and 5% in the esophagus 44. However, primary GIST can also arise in the following uncommon sites other than GI tract: mesentery, omentum, or retroperitoneum 27, and sporadically in the pancreas 42, gallbladder 30, and liver 13. These nongastrointestinal tumors are defined as extragastrointestinal stromal tumors (EGIST).

The GIST arising in liver as a primary lesion is extremely rare, and thus, reports on hepatic GIST and its clinicopathological features as well as clinical outcomes are limited. Therefore, this study was designed to evaluate the clinicopathological features and prognosis of primary hepatic GIST in order to achieve the optimal treatment strategy.

## Materials and Methods

One case of hepatic GIST, which was the only case from May 2010 to March 2015 in our center, and 22 cases of hepatic GIST reported in the literature were enrolled into this study. Literature published from 2001 to 2015 was searched in the databases of MEDLINE and China National Knowledge Infrastructure (CNKI). We found 12 cases of hepatic GIST in English 1, 3, 13, 18, 20, 21, 22, 24, 25, 29, 36, 45 and additional 10 cases in Chinese 2, 12, 14, 23, 31, 38, 39, 40, 41, 43 by literature search. In addition, clinical and pathological characteristics as well as prognosis of hepatic GIST were compared with those of gastric and small intestinal GIST. All 297 cases of gastric GIST and 59 cases of small intestinal GIST were diagnosed and treated in our center from 2001 to 2015. This study was approved by the Ethics Committee of Xijing Hospital, and written informed consents were obtained from the patients.

The following clinical and pathological data were collected: age, gender, symptoms, primary tumor site, density of the tumor, CT enhancement, tumor size, surgical intervention, histological cell types, mitotic index, Ki‐67 expression, gene mutation status, National Institutes of Health (NIH) classification, adjuvant imatinib mesylate therapy, and survival. The GISTs were classified as very low, low, intermediate, and high risk following the modified protocol of NIH risk classification reported by Joensuu et al. 17.

For survival analysis, the exclusion criteria were as follows: (1) GIST in the organs other than hepatic GIST; (2) Patients suffered from other type of malignant tumors in addition to hepatic GIST; (3) Patients had distant metastasis; (4) Patient had tumor rupture during operation; (5) Patient had received neoadjuvant imatinib mesylate therapy; (6) Patient did not receive R0 resection; (7) Patients failed to have follow‐up data.

Data was processed using SPSS 22.0 for Windows (SPSS Inc., Chicago, IL). Numerical variables were expressed as mean ± SD. Discrete variables were analyzed using the chi‐square test or Fisher's exact test. Risk factors for survival were identified by univariate analysis and COX regression was employed for multivariate analysis. Disease‐free survival (DFS) and disease‐specific survival (DSS) were analyzed by the Kaplan–Meier method and differences between the curves were compared using log‐rank test. *P* values were considered to be statistically significant at the 5% level.

## Results

### General features of the hepatic GIST

Clinical and pathological features of hepatic GISTs are summarized in Table**** 1. Of the 23 cases, 12 were male (52.2%) and 11 were female (47.8%), and aged from 17 to 79 years (median, 55 years; mean, 52 years). The most common symptom was abdominal discomfort (12/21, 57.1%), followed by abdominal pain (9/21, 42.9%), abdominal mass (5/21, 23.8%), anorexia (3/21, 14.3%), bleeding or anemia (1/21, 4.8%), weight loss (1/21, 4.8%), and other symptoms including dyspnea, fatigue, constipation, vomiting, and abdominal distension (12/22, 54.5%). Incidence of hepatic GIST was higher in the right lobe (10/21, 47.6%) than that in the left lobe (7/21, 33.3%). Majority of the tumors (11/12, 91.7%) were displayed as low density by regular CT examination, followed by light (7/12, 58.3%), moderate (3/12, 25.0%), or high (2/12, 16.7%) density, respectively. Sixteen patients (16/23, 69.6%) underwent complete surgical resection, six patients (6/12, 50%) received imatinib therapy after surgery, one patient was treated with imatinib therapy only, and one patient received radiofrequency ablation therapy.

**Table 1:** Clinical and pathological characteristics of hepatic GISTs

Characteristics	Number	Percentage (%)
Age (∑=23)		
≤60	14	60.9
>60	9	39.1
Gender (∑=23)		
Male	12	52.2
Female	11	47.8
Symptoms		
Abdominal pain (∑=21)	9	42.9
Bleeding or anemia (∑=21)	1	4.8
Abdominal mass (∑=21)	5	23.8
Anorexia (∑=21)	3	14.3
Abdominal discomfort (∑=21)	12	57.1
Weight loss (∑=21)	1	4.8
Others (∑=22)	12	54.5
Location (∑=21)		
Right lobe	10	47.6
Left lobe	7	33.3
Both lobe	4	19.0
Density of tumor (∑=12)		
Low	11	91.7
Moderate	1	8.3
CT enhancement (∑=12)		
No	0	0
Light	7	58.3
Moderate	3	25.0
High	2	16.7
Tumor size (∑=23)		
0–2 cm	0	0
2–5 cm	1	4.3
5–10 cm	8	34.8
>10 cm	14	60.9
Surgical resection (∑=23)		
Complete resection	16	69.6
Incomplete resection	0	0
No surgery	7	30.4
Morphology (∑=21)		
Spindle	18	85.7
Epithelioid	0	0
Mixed	3	14.3
Mitotic index (∑=16)		
≤5	4	25.0
>5	12	75.0
Ki‐67 positivity (∑=6)		
<5	0	0
>5	6	100
Immunohistochemistry		
CD117 positivity (∑=23)	23	100
CD34 positivity (∑=19)	11	57.9
DOG‐1 positivity (∑=6)	5	83.3
Vimentin positivity (∑=10)	9	90.0
S‐100 positivity (∑=18)	0	0
SMA positivity (∑ = 17)	5	29.4
Genomic mutation (∑ = 4)		
*KIT*	2	50.0
*PDGFRA*	0	0
Wild type	2	50.0
NIH risk category (∑ = 22)		
Very low risk	0	0
Low risk	0	0
Intermediate risk	0	0
High risk	22	100
Adjuvant therapy (∑=12)		
Yes	6	50.0
No	6	50.0

GIST, gastrointestinal stromal tumor; CT, computed tomography; NIH, National Institutes of Health; KIT, c‐kit proto‐oncogene protein; PDGFRA, platelet‐derived growth factor receptor α.

Size of the tumors ranged from 4.3 to 44.0 cm in diameter (median, 15.0 cm; mean, 14.6 cm). Mitotic index was over 5/50 HPF in 12 out of 16 patients (75.0%) and Ki‐67 expression was detected at least in 5% of the cells in all six of the patients (100%) who received Ki‐67 examination. Out of 25 hepatic GIST specimens, spindle cell morphology was observed in 18 (85.7%) of them, and mixed morphology in three of the tumor specimens (12.0%) was observed.

Positive CD117 expression in 23 out of 23 (100%) specimens, positive CD34 expression in 11 out of 19 (57.9%) specimens, positive DOG‐1 expression in five of six (83.3%) specimens, positive expression of vimentin in nine out of 10 (90.0%), and positive SMA expression in five out of 17 (29.4%) specimens were observed. Genomic mutation was examined in four specimens, and *KIT* mutation at exon 11 was found in two of the four specimens, while the rest two specimens were without any significant gene mutation. Twenty‐two patients were classified as high risk (22/22, 100%) by the NIH risk classification.

### Survival of hepatic GIST

Survival data of hepatic GISTs were analyzed and summarized in Table**** 2. By the inclusion/exclusion criteria described in the methods, the survival rate was analyzed in 11 hepatic GIST patients with range of follow‐up from 4 to 108 months (mean, 27.1 months; median, 13 months). Of the 11 cases, two patients had recurrent hepatic GIST and two patients had metastatic GIST in the organs other than liver, three patients died from hepatic GIST. The 5‐year median DFS time was 24 months and 5‐year DSS rate was 33.3%, respectively.

**Table 2:** Survival data of hepatic GISTs (*N *=* *11)

Observation	Result
Follow‐up time (month)	
Mean ± SD	27.1 ± 30.6
Median (range)	13.0 (4.0, 108.0)
Survival data	
Recurrence	2
Metastasis	2
GISTs‐related death	3
5‐year median DFS time (months)	24.0
5‐year DSS rate (%)	33.3

SD, standard deviation; DFS, disease‐free survival; DSS, disease‐specific survival.

### Comparison among hepatic, gastric, and small intestinal GIST

Next, clinical and pathological feature of 23 hepatic GISTs were compared with that of 297 gastric GISTs and 59 small intestinal GISTs (Table**** 3). The results showed that tumor size, mitotic index, NIH risk category, and adjuvant therapy were significantly different between hepatic and gastric GISTs (all *P *<* *0.05), that is, incidence of tumors with larger size or high‐risk tumors was significantly higher in hepatic GIST group compared to that in gastric GIST group. Hepatic GIST group also showed larger size, higher mitotic index, and higher risk category of NIH in comparison with those of small intestinal GIST group (All *P *<* *0.05).

**Table 3:** Comparison of selected clinicopathological parameters among hepatic, gastric, and small intestinal GISTs

Characteristics	Liver(N = 23)	Stomach		Small intestine	
		(N = 297)	*P*‐value	(N = 59)	*P*‐value
Age			0.688		0.877
≤60	14 (60.9%)	168 (56.6%)		37 (62.7%)	
>60	9 (39.1%)	129 (43.4%)		22 (37.3%)	
Gender			0.999		0.557
Male	12 (52.2%)	155 (52.2%)		35 (59.3%)	
Female	11 (47.8%)	142 (47.8%)		24 (40.7%)	
Tumor size			<0.001		<0.001
≤2 cm	0	96 (32.3%)		4 (6.9%)	
2.1–5 cm	1 (4.3%)	107 (36.0%)		26 (44.8%)	
5.1–10 cm	8 (34.8%)	72 (24.2%)		17 (29.3%)	
>10 cm	14 (60.9%)	22 (7.4%)		11 (19.0%)	
Morphology			0.222		0.696
Spindle	18 (85.7%)	275 (92.6%)		51 (89.5%)	
Epithelioid/mixed	3 (14.3%)	22 (7.4%)		6 (10.5%)	
Mitotic index			0.020		0.043
≤5	4 (25.0%)	163 (54.9%)		29 (53.7%)	
>5	12 (75.0%)	134 (45.1%)		25 (46.3%)	
NIH risk category			<0.001		0.003
Very low	0	83 (27.9%)		4 (7.4%)	
Low	0	58 (19.5%)		17 (31.5%)	
Intermediate	0	87 (29.3%)		0	
High	22 (100%)	69 (23.2%)		33 (61.1%)	
Adjuvant therapy			0.044		0.531
Yes	6 (50.0%)	68 (23.1%)		36 (61.0%)	
No	6 (50.0%)	226 (76.9%)		23 (39.0%)	

NIH, National Institute of Health.

**Figure 1:**
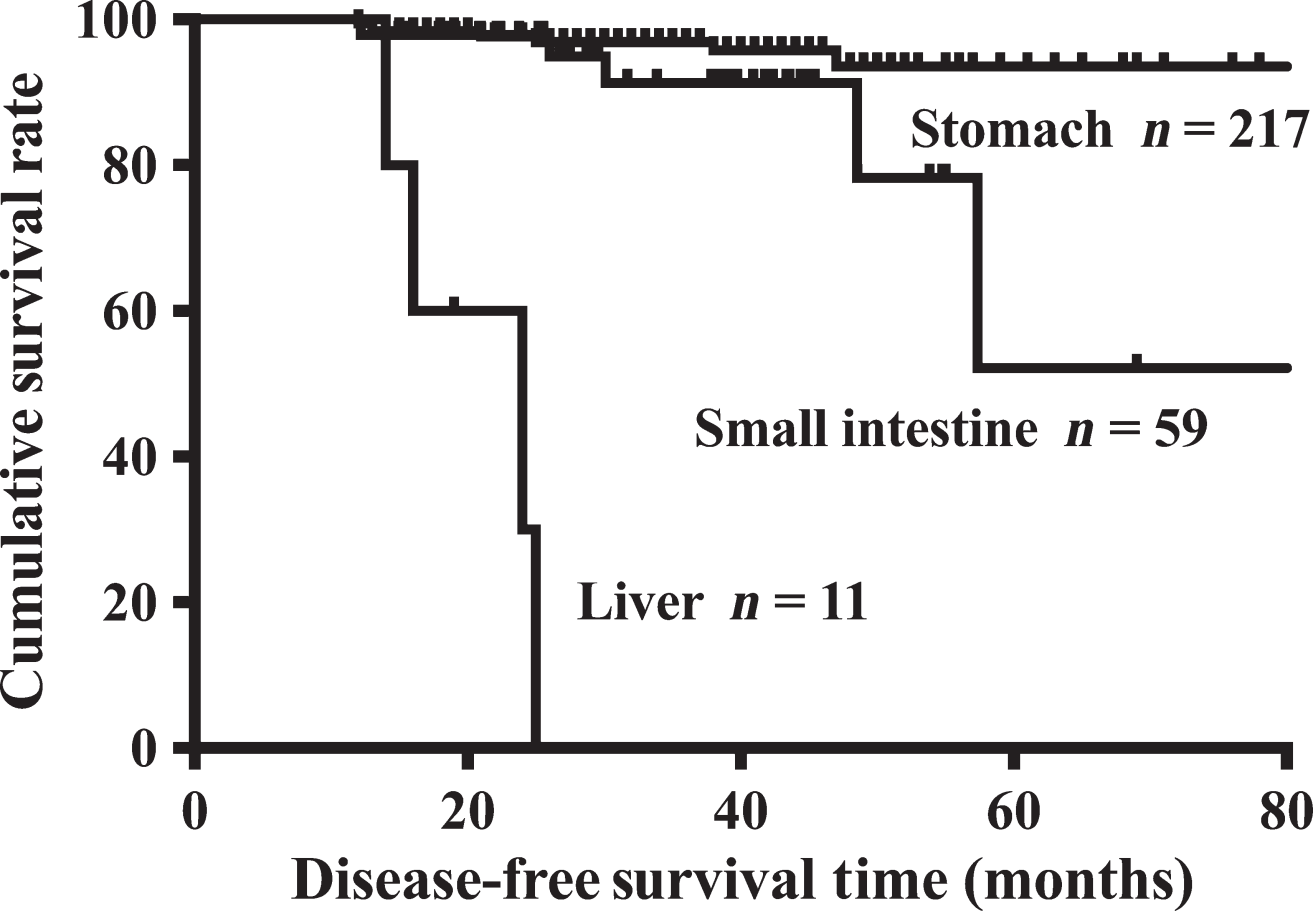
Comparison of disease‐free survival (DFS) between hepatic, gastric, and small intestinal GISTs. Liver versus Stomach: *P *<* *0.001; Liver versus Small intestine: *P *<* *0.001. Vertical axes: percent of survival; horizontal axes: time (months). GIST, gastrointestinal stromal tumor.

**Figure 2:**
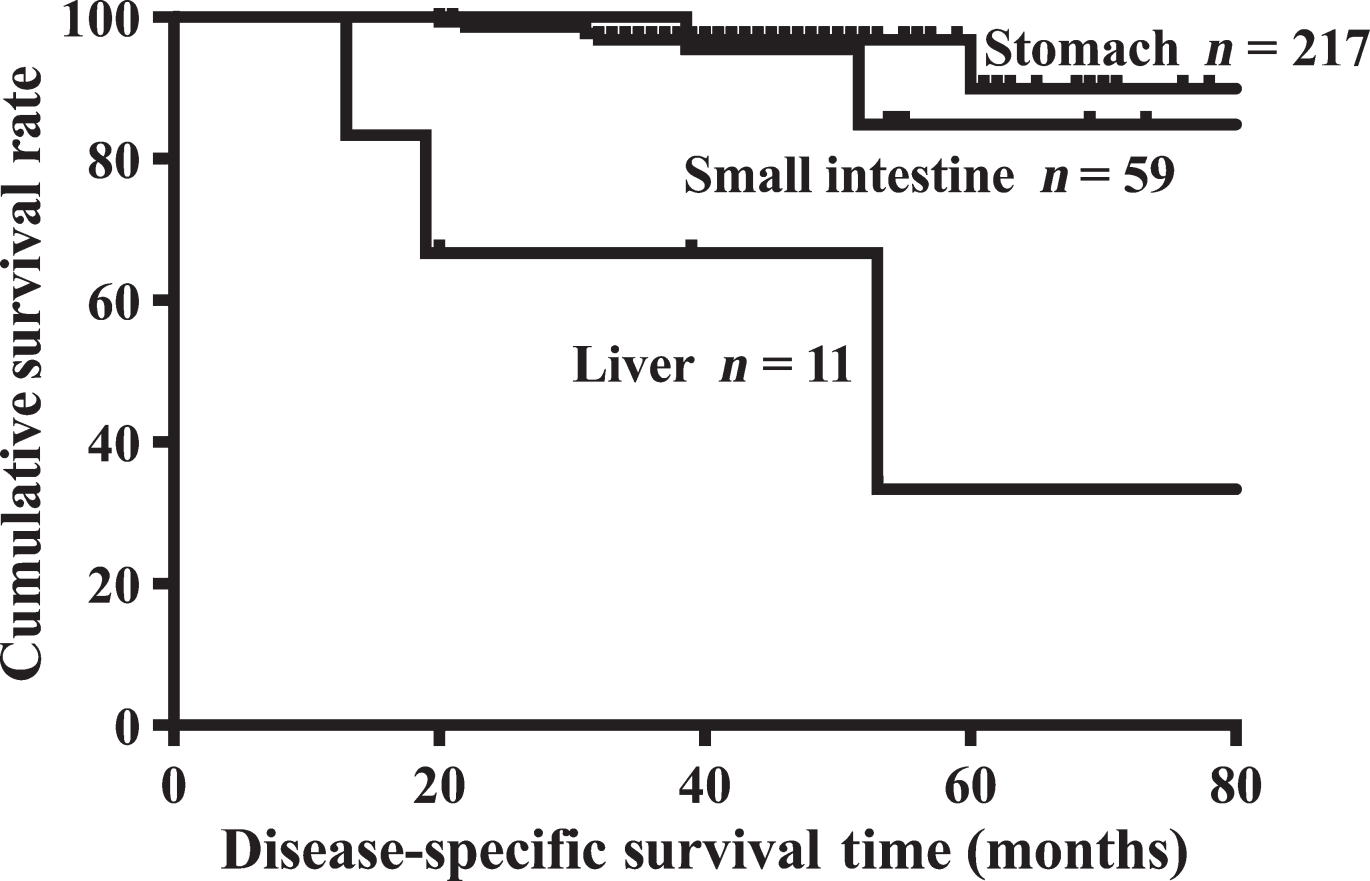
Comparison of disease‐specific survival (DSS) between hepatic, gastric, and small intestinal GISTs. Liver versus Stomach: *P *<* *0.001; Liver versus Small intestine: *P *=* *0.004. Vertical axes: percent of survival; horizontal axes: time (months). GISTs, gastrointestinal stromal tumors

In order to analyze the prognosis among hepatic, gastric, and small intestinal GISTs, survivals of 11 hepatic GISTs were compared to those of 217 gastric GISTs and 59 small intestinal GISTs which were enrolled in our center and have complete follow‐up data. The results showed that the DFS (5‐year median survival time: 24 months vs. 25 months, *P *<* *0.001, Fig.**** 1) and DSS (5‐year survival rate: 33.3% vs. 89.9%, *P *<* *0.001, Fig.**** 2) of hepatic GISTs were significantly worse than those of gastric GISTs. The DFS (5‐year median survival time: 24 months vs. 30 months, *P *<* *0.001, Fig.**** 1) and DSS (5‐year survival rate: 33.3% vs. 84.8%, *P *=* *0.004, Fig.**** 2) of hepatic GISTs were significantly worse than those of small intestinal GISTs. Furthermore, univariate and multivariate analysis were performed to evaluate the prognostic value of location (Table**** 4, 5). The results showed that location was an independent prognostic factor for DFS (stomach vs. liver: *P *=* *0.003; small intestine vs. liver: *P *=* *0.007) of GIST patients. For prognosis of DSS, the location between the stomach and liver was not an independent risk factor (*P *=* *0.096), while the location was an independent risk factor for small intestine and liver (*P *=* *0.040).

**Table 4:** Univariate analysis of variables associated with DFS and DSS in patients with hepatic, gastric, and small intestinal GISTs

Characteristics	β	Hazard ratio (95% CI)	*P*‐value
DFS			
Age	−0.727	0.483 (0.156–1.502)	0.209
Gender	−0.437	0.646 (0.234–1.778)	0.397
Tumor size	2.110	8.251 (3.611–18.851)	<0.001
Morphology	1.092	2.981 (0.662–13.416)	0.155
Mitotic index	1.521	4.579 (1.270–16.504)	0.020
NIH risk category	1.723	5.600 (1.983–15.816)	0.001
Adjuvant therapy	1.730	5.638 (1.947–16.331)	0.001
Location			<0.001
Stomach vs. liver	−3.979	0.019 (0.005–0.076)	<0.001
Small intestine vs. liver	−2.928	0.054 (0.012–0.233)	<0.001
DSS			
Age	0.912	2.488 (0.696–8.889)	0.161
Gender	0.164	1.179 (0.339–4.098)	0.796
Tumor size	0.783	2.188 (1.072–4.466)	0.031
Morphology	1.734	5.663 (1.164–27.543)	0.032
Mitotic index	2.305	10.020 (1.244–80.697)	0.030
NIH risk category	1.419	4.131 (1.213–14.068)	0.023
Adjuvant therapy	0.600	1.822 (0.508–6.538)	0.357
Location			0.001
Stomach vs. liver	−2.683	0.068 (0.016–0.296)	<0.001
Small intestine vs. liver	−2.337	0.097 (0.016–0.589)	0.011

DFS, disease‐free survival; DSS, disease‐specific survival; NIH, National Institute of Health.

**Table 5:** Multivariate analysis of prognostic factors for DFS and DSS in patients with hepatic, gastric, and small intestinal GISTs

Characteristics	β	Hazard ratio (95% CI)	*P*‐value
DFS			
Tumor size	1.760	5.811 (2.325–14.524)	<0.001
Location			0.007
Stomach vs. liver	−2.336	0.097 (0.020–0.457)	0.003
Small intestine vs. liver	−2.181	0.113 (0.023–0.548)	0.007
DSS			
Age	1.437	4.208 (0.953–18.585)	0.058
NIH risk category	1.366	3.918 (1.077–14.254)	0.038
Location			0.078
Stomach vs. liver	−1.317	0.268 (0.057–1.261)	0.096
Small intestine vs. liver	−2.397	0.091 (0.009–0.901)	0.040

DFS, disease‐free survival; DSS, disease‐specific survival; NIH, National Institute of Health.

## Discussion

In this study, we summarized clinical and pathological features of 23 cases of hepatic GIST. Of the 23 cases, one case was diagnosed and treated in our center and the rest 22 cases were literature searched in MEDLINE for English and CNKI for Chinese publications. We further analyzed prognosis of hepatic GIST in comparison with that of gastric and small intestinal GIST. It was found that most common clinical symptoms for hepatic GIST were abdominal discomfort and abdominal pain, majority of the tumors were low density by CT examination, and fibroblast‐like spindle cell shape was predominant by histology. In addition, hepatic GIST had poorer prognosis compared to gastric GIST and small intestinal GIST.

It has been reported that GIST is considered to originate from interstitial cells of Cajal (ICC), the pacemaker of gastrointestinal tract 19. Furthermore, a subset of “ICC‐like” interstitial cells were observed in organs outside of the gastrointestinal tract, which was similar in structure and function to ICCs 16. Recently, the existence of intrahepatic ICCs in the portal spaces and septa was demonstrated through the immunohistochemistry in human specimens 33. Additionally, Rusu et al.^32^ found the evidence that ICCs also existed in human embryonic liver presented as the distinctively precursor/progenitor cells. While the distribution of ICCs in the liver remains to be defined, existence of ICCs in hepatic tissue may contribute to the development of hepatic GISTs. In this study, the incidence of GIST in each lobe was comparable indicating ICCs may exist in both lobes. However, the role of ICCs in the development of GIST remains to be further investigated.

Previous studies indicated that liver is the most popular organ for metastasis of GIST originated from gastrointestinal tract 5. Size of the metastatic GIST in liver is usually large and often found in both lobes 46. In fact, CT findings of metastatic GIST are similar to those of primary GIST 11. Vanel et al.^37^ reported that the imaging feature of liver metastatic GIST was heterogeneous hypodense lesions with progressive and concentric enhancement. In this study, majority of the primary hepatic GISTs were in large size, comparably found in both lobes, and showed as low density with variety degrees of enhancement on image examination, which was similar to the feature of liver metastatic GISTs as described above. Thus, differential diagnosis of primary and metastatic hepatic GISTs is difficult, but it is important to differentiate them from the point view of therapy. In this regard, imaging examinations including computed tomography (CT), ultrasound (US), esophagogastroduodenoscopy (EGD), and colonoscopy are generally used to differentiate the primary and metastatic liver GISTs. However, Luo et al. (Miettinen et al.) reported discrepancies of contrast‐enhanced ultrasound (CEUS) and enhanced CT examination findings in the distinct vascular architecture of primary and metastatic liver GISTs. Thus, intraoperative inspection is also often applied to confirm origination of GISTs 10, 26.

Preoperative diagnosis of extragastrointestinal stromal tumors (EGISTs) is also relatively difficult due to the deeper location and lack of mucosal connection, which could potentially lead to misdiagnosis 8. In this content, differential diagnosis of GISTs in liver may involve the poorly differentiated carcinomas, epithelioid angiomyolipoma and leiomyosarcoma, or malignant melanoma 44. In these instances, the ultrasound‐guided fine needle aspiration biopsy (US‐FNAB) ought to be performed in order to make a definite diagnosis after which different treatment strategies will be applied to primary or metastatic liver GISTs.

Due to variety kinds of clinical presentation of the GIST, treatment and prognosis of this tumor is variable 4, 7. It has been reported that approximately 10%–30% of GISTs were regarded as clinically malignant 15. In this study, clinical and pathological characteristics of hepatic GISTs were analyzed in comparison with gastric GISTs. It was found that tumor size and NIH risk category were significantly higher in hepatic GISTs than that in gastric GISTs. While it has also been reported that tumor size and mitotic index are the most efficient prognostic factors in determining malignancy of GISTs 4, this study, could not predict the survival rate from tumor size and mitotic index due to the limit of sample size of hepatic GISTs.

Original site of a primary GIST is also an independent predictor for the prognosis of GISTs 34. In the NIH risk classification system, GIST is classified as gastric or nongastric GIST, and hepatic GIST is not included yet. Thus, we compared the prognosis of hepatic GISTs with gastric and small intestinal GISTs from our center. The results showed that the DFS and DSS of hepatic GISTs were significantly worse than those of gastric and small intestinal GISTs. However, the multivariate analysis showed that location was an independent prognostic factor for DFS (stomach vs. liver; small intestine vs. liver) of GIST patients. For prognosis of DSS, the location between small intestine and liver was an independent risk factor, while the location was not an independent risk factor for stomach and liver. This contrary results of DSS may attribute to the limitation of our sample size and the less tumor‐related death of GISTs. Furthermore, it is unavoidable that the low incidence of adjuvant therapy of Imatinib in this study would lead to bias during the survival analysis. Thus, the actual prognosis of hepatic GISTs may be more favorable than that in this study.

There are some limitations in this study. First, this study is a retrospective analysis and lacks systematic and prospective data. Second, sample size of the hepatic GIST was small. Third, due to the limited number of duodenal or rectal GIST cases in our center, these types of GISTs were not included in this study.

## Conclusions

Majority of the primary hepatic GISTs are large in size and highly malignant. Clinical and pathological features of hepatic GIST are significantly different from that of gastric and small intestinal GIST. Prognosis of the primary hepatic GISTs is very poor and worse than that of gastric and small intestinal GISTs.

## Conflict of interests

There are no financial or other relations that could lead to a conflict of interest.

## Supporting information

Table S1: Clinicopathological features of hepatic GISTs
